# Efficacy and safety of extended use of platinum-based doublet chemotherapy plus endostatin in patients with advanced nonsmall cell lung cancer

**DOI:** 10.1097/MD.0000000000004183

**Published:** 2016-07-18

**Authors:** Weiheng Hu, Jian Fang, Jun Nie, Ling Dai, Jie Zhang, Xiaoling Chen, Xiangjuan Ma, Guangming Tian, Di Wu, Sen Han, Jindi Han, Yang Wang, Jieran Long

**Affiliations:** Key Laboratory of Carcinogenesis and Translational Research (Ministry of Education/Beijing), Department of Thoracic Oncology II, Peking University Cancer Hospital and Institute, Beijing, China.

**Keywords:** chemotherapy, endostatin, extended therapy, NSCLC

## Abstract

The aim of this study was to investigate the efficacy and safety of the extended use of platinum-based doublet chemotherapy (PT-DC) plus endostatin in patients with advanced nonsmall cell lung cancer (NSCLC).

We performed a retrospective analysis of 200 newly diagnosed advanced NSCLC patients who had received at least 1 cycle of endostatin plus PT-DC between September 2009 and November 2014. Of these patients, 155 received 4 or more cycles of therapy (the extended therapy group), while 45 received less than 4 cycles of therapy (the control group). Clinical tumor responses, progression-free survival (PFS), overall survival (OS), and toxicity profiles were recorded and retrospectively analyzed.

In the extended therapy group, 67 patients (43.2%) achieved a best overall response rate of partial response (PR), while in the control group, 13 patients (28.9%) had a best overall response rate of PR. After a median follow-up of 15.9 months, the median PFS and OS were 8.0 and 23.1 months in the extended arm and 5.8 and 14.0 months in the control arm, respectively. There were statistically significant differences in median PFS and OS between these 2 arms. Hematologic and gastrointestinal toxicities occurred more frequently in the extended therapy group, but no statistically significant difference was detected in grade 3 to 4 toxicities overall between these 2 groups.

In conclusion, extended treatment using endostatin combined with PT-DC can provide additional survival benefits and satisfactory toxicity profiles in previously untreated patients with NSCLC, which merits further evaluation in a larger prospective study.

## 1 Introduction

Lung cancer is the leading cause of cancer-related deaths worldwide. Approximately 85% of lung cancer patients have nonsmall cell lung cancer (NSCLC), and nearly two-thirds of them have already reached an advanced stage (stage IIIB/IV) by the time of diagnosis, which always following with bad prognosis although treated with improved therapies.^[1–3]^ According to current guidelines, the first-line treatment strategy for advanced NSCLC should take into account age, histology, molecular pathology, comorbidities, and the performance status of patients, and platinum-based doublet chemotherapy (PT-DC) has been recommended as the standard first-line treatment for such individuals, especially those without epidermal growth factor receptor (EGFR) mutations.^[4–6]^ However, recent studies have revealed that the efficacy of such regimens has reached a plateau, which presents an urgent need for the development of new therapeutic combinations with improved efficacy and acceptable toxicity.^[7,8]^

It has been well documented that angiogenesis plays a central role in the process of tumor growth and metastatic dissemination.^[9]^ Over the past decade, the development of antiangiogenic agents have grown remarkably and become one of the most promising areas in cancer medicine.^[10]^ Currently, several antiangiogenic agents have been approved for the treatment of patients with advanced NSCLC. For example, bevacizumab, a recombinant humanized antivascular endothelial growth factor monoclonal antibody, is the first antiangiogenic therapy approved by the Food and Drug Administration (FDA). The E4599 and AVAiL trials have shown that bevacizumab, in combination with PT-DC as a first-line therapy, significantly improves survival outcomes in patients with advanced nonsquamous NSCLC.^[11,12]^ More importantly, in a recently published clinical study, the continuation of bevacizumab plus standard chemotherapy beyond disease progression was demonstrated to provide further survival benefits in patients with metastatic colorectal cancer.^[13]^ On the basis of these results, continued antiangiogenesis by bevacizumab appears to be necessary in order to maximize the clinical benefits derived from initial therapy, and this finding may be extrapolated to other antiangiogenic agents.

Endostatin is a novel potent inhibitor of angiogenesis, which has been developed and approved in China. A number of studies, both in vitro and in vivo, have demonstrated that endostatin combined with concurrent chemoradiotherapy has a broad spectrum of activity against solid tumors, such as breast cancer, metastatic melanoma as well as NSCLC, with a favorable toxicological profile.^[14–16]^ A recent meta-analysis study showed that in patients with advanced NSCLC, the addition of endostatin to platinum-based chemotherapy resulted in a significantly higher efficacy and similar tolerability as compared with chemotherapy alone.^[17]^ However, the optimal duration of chemotherapy combined with endostatin remains uncertain, and whether the continued use of endostatin could help to maximize the clinical benefits derived from initial therapy is also unknown. To address these issues, in the current study, we retrospectively investigated the efficacy and safety of extended use of endostatin plus PT-DC (4 or more cycles) in the treatment of advanced NSCLC.

## 2 Patients and methods

### 2.1 Patients

A total of 272 consecutive advanced NSCLC patients who received at least 1 cycle of endostatin plus PT-DC at the authors’ institution between September 2009 and November 2014 were screened for this retrospective study. The inclusion criteria were as follows: histologically or cytologically confirmed stage IIIB or IV NSCLC; newly diagnosed without prior treatment history; having measurable lesions (according to the Response Evaluation Criteria in Solid Tumors); and having adequate bone marrow, hepatic, and renal function. The exclusion criteria were: individuals with rapid disease progression or intolerable toxicity during the treatment course; having major organ dysfunction or other serious complications; pregnant or breast feeding women; history or presence of clinically significant cardiovascular diseases; and incomplete clinical data. The study protocol was approved by the ethical review board of the authors’ institution prior to initiation.

### 2.2 Treatment regimens

The PT-DC regimens used for our patients included pemetrexed (PEM, Alimta, Eli Lilly, Indianapolis, IN) and cisplatin (DDP, Qilu Pharmaceutical Co. Ltd, Jinan, China), gemcitabine (GEM, Gemzar, Eli Lilly) and DDP, irinotecan (CPT-11, Camptosar, Pfizer, New York, NY) and DDP, PEM and nedaplatin (NDP, Simcere Pharmaceutical Groups, Nanjing, China), and GEM and NDP. PEM was administered intravenously (via IV) at 500 mg/m^2^ on day 1; DDP and NDP were administered via IV at 75 mg/m^2^ on day 1; GEM was administered via IV at 1250 mg/m^2^ on days 1 and 8; and CPT-11 was administered via IV at 60 mg/m^2^ on days 1, 8, and 15. Endostatin (Endostar, Simcere Pharmaceutical Groups) was infused via IV at 7.5 mg/m^2^ once daily for 14 days along with PT-DC therapy. Cycles were repeated every 21 days (PEM/GEM plus DDP/NDP) or 28 days (CPT-11 plus DDP) until disease progression or unacceptable toxicity were reached. Patients who received 4 or more cycles of therapy were designated as the extended therapy group, while those who received less than 4 cycles of therapy were designated as the control group. During the treatment, concurrent radiotherapy was performed based on the judgments of the treating physicians. For patients who developed progression, EGFR tyrosine kinase inhibitors (TKIs), or docetaxel-based chemotherapy were used for subsequent treatment.

### 2.3 Assessment of efficacy and toxicity

Clinical data were retrospectively collected from the medical records of all participants. Tumor response was evaluated as complete response, partial response (PR), stable disease, or progressive disease in accordance with the Response Evaluation Criteria in Solid Tumors version1.1 every 2 cycles or when required for radiologic examinations.^[18]^ Progression-free survival (PFS) was calculated from the date of initiation of treatment to the date of disease progression or death due to any cause. Overall survival (OS) was measured from the date of treatment initiation to death from any cause. PFS and OS were censored at the last follow-up date if the event had not occurred. The best overall response was defined as the best response recorded from the start of treatment until disease progression or withdrawal from treatment. Toxicity assessment was performed for all patients. Adverse events and laboratory abnormalities were assessed weekly according to the Common Terminology Criteria for Adverse Events of the National Cancer Institute (CTCAE version4.0).

### 2.4 Statistical data analysis

Statistical analysis was carried out using SPSS 19.0 software (IBM Corp., Armonk, NY). Categorical data are reported as counts and percentages, whereas continuous data are presented as medians and ranges. The Chi-squared test or Fisher exact test was used to compare categorical data. PFS and OS were calculated according to the Kaplan–Meier method, and differences between survival curves were estimated using the log-rank test. A multivariable Cox proportional hazards regression model was used to calculate hazard ratios (HRs) and 95% confidence intervals. A *P*-value of less than 0.05 was considered statistically significant.

## 3 Results

### 3.1 Patient demographics

A total of 155 NSCLC patients who received extended use of PT-DC plus endostatin and met the inclusion criteria were enrolled in this study and served as the extended therapy group. In addition, 45 NSCLC patients who were treated with 3 or less cycles of PT-DC plus endostatin were included as controls. The demographics of the 2 groups are summarized in Table 1. Both treatment groups were well balanced in terms of patient and disease characteristics, except that the extended therapy group had a higher proportion of patients with a smoking history and stage IV disease. Furthermore, in the extended therapy group, 101 patients (65.2%) received 4 cycles of PT-DC plus endostatin, 40 (25.8%) received 5 or 6 cycles, and 14 (9.0%) received 7 or more cycles; whereas in the control group, 11 (24.4%) received 1 cycle of chemotherapy, 25 (55.6%) received 2 cycles, and 9 (20.0%) received 3 cycles.

**Table 1:**
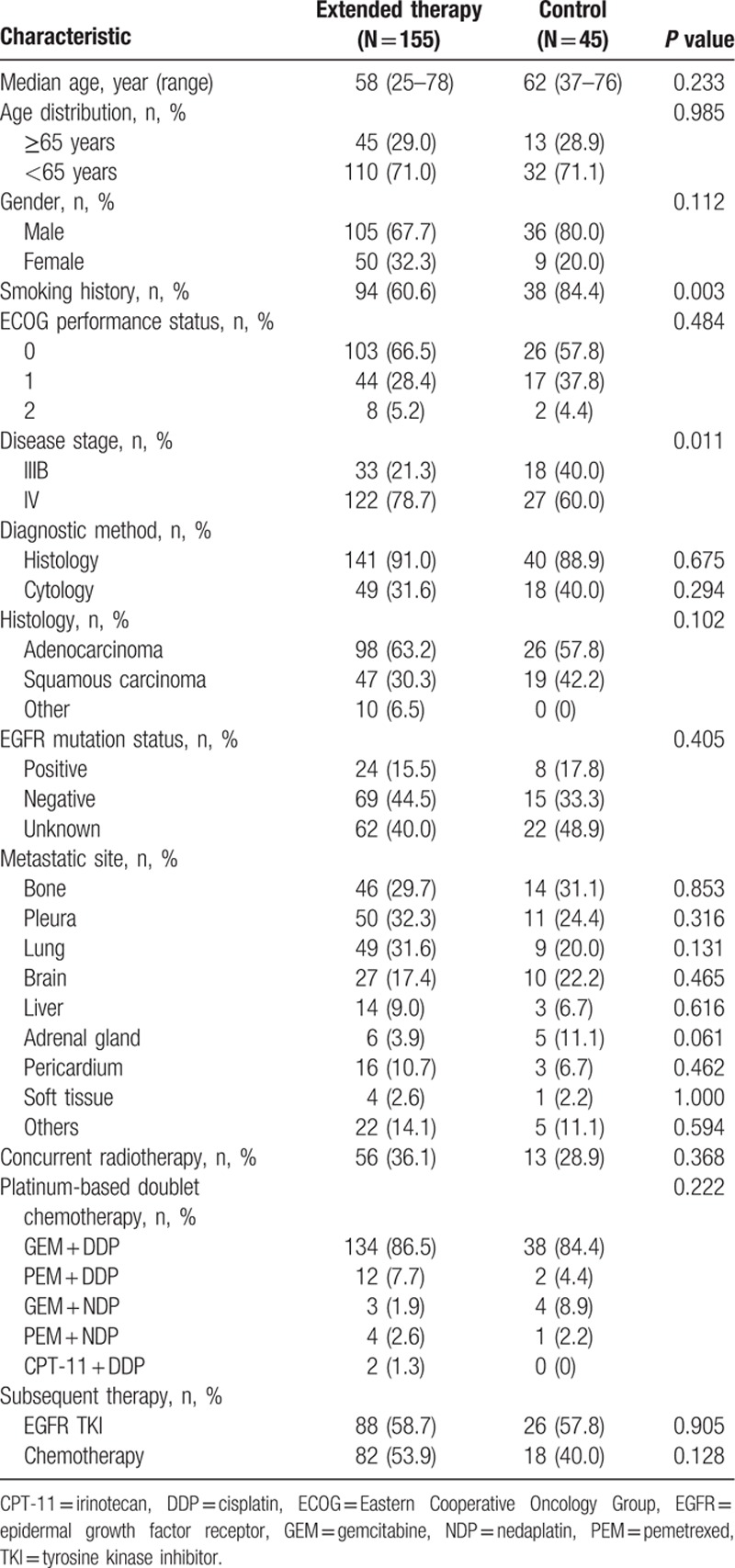
Baseline patient characteristics.

### 3.2 Response and survival assessment

**Figure 1:**
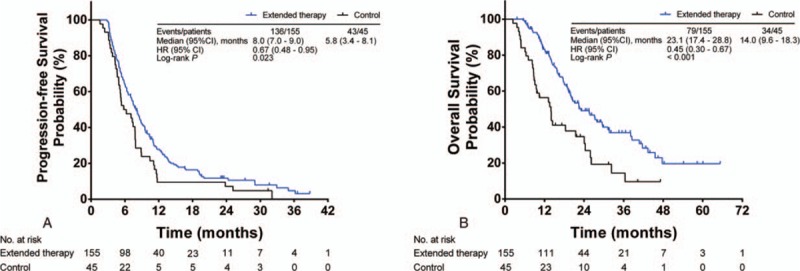
Kaplan–Meier estimated survival for 155 patients in the extended therapy group (blue line) and 45 in the control group (black line). (A) PFS, (B) OS. CI = confidence interval, HR = hazard ratio, OS = overall survival, PFS = progression-free survival.

The best overall responses to treatment in the 2 study groups are summarized in Table 2. In the extended therapy group, 67 patients (43.2%, 67 out of 155) achieved a best overall response of PR; while in the control group, less than one-third of patients (28.9%, 13 out of 45) had a best overall response of PR. After a median follow-up time of 15.9 months (range: 2.4–65.3 months), 113 patients (56.5%, 113 out of 200) had died of NSCLC-related causes, 79 (51.0%, 79 out of 155) of which were in the extended therapy group while 34 (75.6%, 34 out of 45) were in the control group. The Kaplan–Meier curves of PFS and OS for both groups are shown in Fig. 1A and B. The median PFS and OS were 8.0 and 23.1 months in the extended therapy arm, and 5.8 and 14.0 months in the control arm, respectively. There were statistically significant differences in median PFS and OS between the 2 arms (*P* = 0.023 for median PFS and *P* < 0.001 for median OS, respectively), indicating that extended use of PT-DC plus endostatin resulted in a substantial survival advantage for NSCLC patients (HR = 0.67 [95% confidence interval = 0.48–0.95] for PFS, and HR = 0.45 [0.30–0.67] for OS). In addition, multivariate analysis for the extended therapy group showed that disease stage, baseline performance status, EGFR mutation status, and subsequent EGFR-TKI therapy were all significant independent predictors for PFS and OS (data not shown).

**Table 2:**
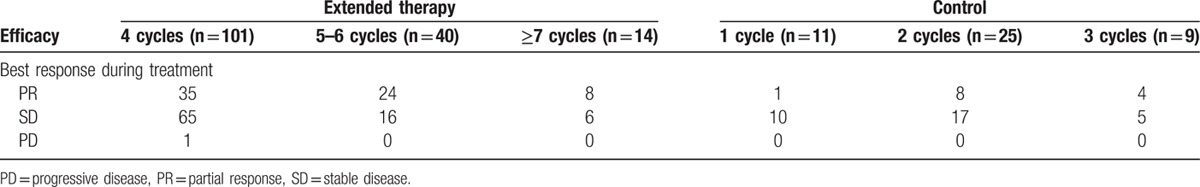
Best responses to treatment in the 2 study groups.

**Figure 2:**
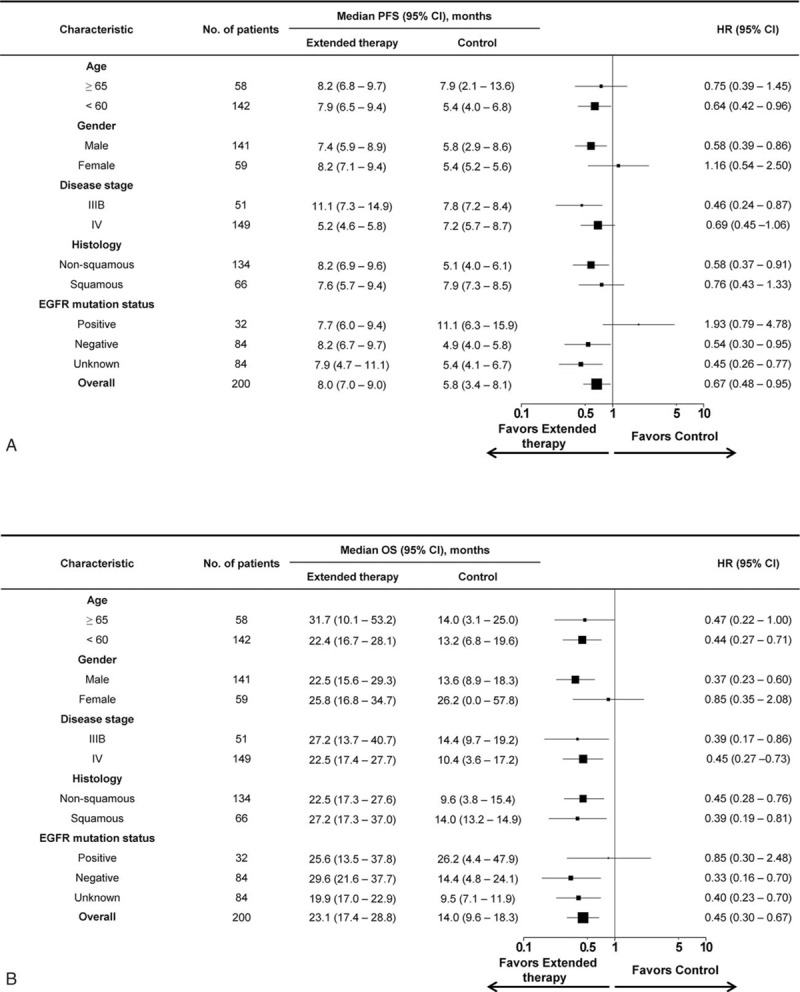
Forest plot for subgroup analyses of PFS (A) and OS (B). Treatment with extended use of PT-DC plus endostatin resulted in a survival benefit in most patient subgroups. Size of the square is proportional to the precision of the study-specific effect estimates, and the bars indicate the corresponding 95% CIs. CI = confidence interval, EGFR = epidermal growth factor receptor, HR = hazard ratio, OS = overall survival, PFS = progression free survival, PT-DC = platinum-based doublet chemotherapy.

Furthermore, we performed a subgroup analysis using a Cox proportional hazard model to determine the association between each demographic variable and the survival benefit. As shown in Fig. 2A and B, the results indicate that, aside from female patients and those with EGFR mutation, substantial PFS and OS benefits were seen in all subgroups after extended use of PT-DC plus endostatin.

### 3.3 Safety

Treatment-related toxicity profiles of the 2 study groups are summarized in Table 3. For both arms, hematologic and gastrointestinal toxicities were the most common adverse events. Patients in the extended therapy group experienced significantly higher rates of leucopenia, neutropenia, anemia, nausea, vomiting, and fatigue, as compared with controls. However, aside from grade 3 to 4 infection, there were no statistically significant differences in grade 3 to 4 toxicities overall between the 2 treatment groups. In addition, it should be noted that 2 patients in the extended therapy group and 3 in the control group experienced grade 3 to 4 cardiac disorders. Most of these disorders occurred in the first or second cycle of therapy, and disappeared following a temporary cessation of therapy or adjustment of the infusion rate.

**Table 3:**
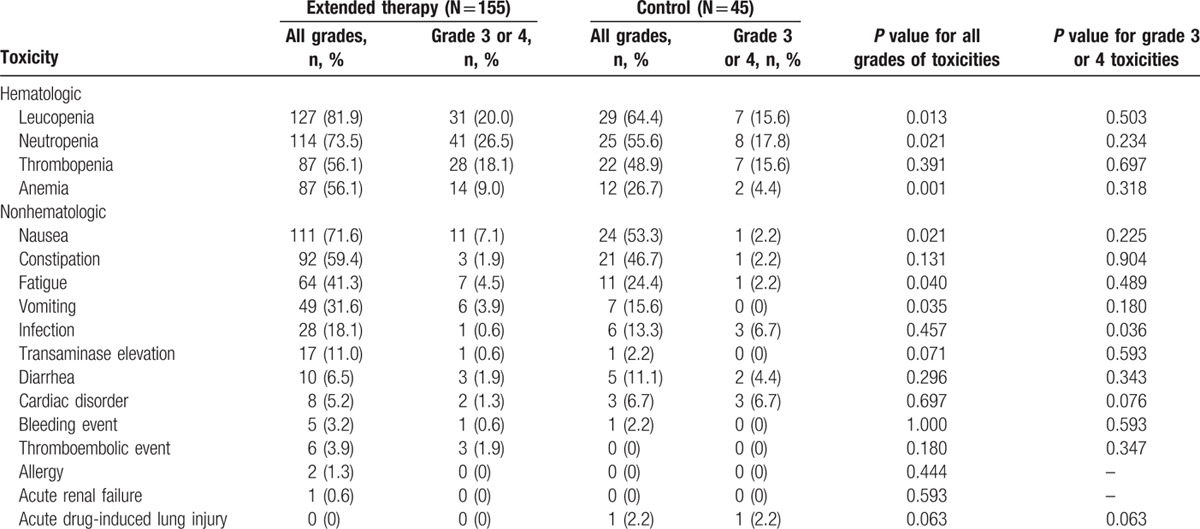
Treatment-related toxicities that occurred in both treatment groups.

## 4 Discussion

The role of platinum-based chemotherapy in patients with advanced NSCLC has been established for 2 decades.^[19]^ In recent years, antiangiogenic agents have also shown efficacy in multiple solid tumors and may provide additional benefits for advanced NSCLC patients receiving a standard first-line platinum-based regimen.^[20]^ Several clinical studies have demonstrated the efficacy and safety of bevacizumab, a humanized antiangiogenic monoclonal antibody, when combined with platinum-based chemotherapy in patients with NSCLC. For example, in the E4599 study, addition of bevacizumab to a paclitaxel/carboplatin regimen is associated with increased survival rates in previously untreated patients with nonsquamous NSCLC.^[11]^ More importantly, the continuation of bevacizumab plus standard chemotherapy beyond disease progression has been demonstrated to provide further survival benefits in patients with metastatic colorectal cancer.^[13]^ This approach is also being tested as a treatment for advanced NSCLC.^[21,22]^ Collectively, these reports support the hypothesis that continued the use of antiangiogenic agents may be necessary to maximize the initial clinical benefits derived from combined therapy with antiangiogenic and chemotherapeutic drugs for lung cancer patients.

As a novel antiangiogenic agent, endostatin has been approved in China for the treatment of NSCLC.^[23]^ Meta-analysis studies reveal that in patients with advanced NSCLC, the combination of endostatin and PT-DC can achieve higher ORR, DCR, and TTP as compared with PT-DC alone.^[17,24]^ Nevertheless, in a multicenter randomized phase II study involving 126 advanced NSCLC patients, the addition of endostatin to a paclitaxel–carboplatin regimen could not significantly improve PFS or OS.^[25]^ These discrepant findings may be due to the limited administration of endostatin therapy (for no more than 3 cycles) in the enrolled patients, thus indicating a rationale for the continued use of endostatinin order to achieve its full clinical benefits. In the current study, we found that advanced NSCLC patients who received ≥4 cycles of endostatin plus PT-DC had significantly longer PFS and OS than those who received <4 cycles of therapy, which points to the importance of sustained suppression of angiogenesis by endostatin in the treatment of advanced NSCLC.

An interesting finding of this study was that extended therapy achieved a more pronounced improvement in OS than in PFS. Previous studies regarding other antiangiogenic agents (e.g., bevacizumab) have demonstrated that discontinuation of antiangiogenic therapy neither accelerates tumor growth nor promotes the development of a more aggressive phenotype in various solid tumors.^[26,27]^ In this study, for patients who received extended therapy, continued suppression of angiogenesis by endostatin, could inhibit further growth and spread of tumor cells. After cessation of endostatin, new vessels began to grow, thereby signaling the creation of an environment supportive to tumor growth (indicated by disease progression).^[28,29]^ Despite such conditions, there may still be a suppressive effect on the induction of a more aggressive tumor phenotype, thus contributing to the persistent OS benefit. The molecular mechanism underlying this phenomenon, we assume, might be related to the complex changes in the tumor microenvironment elicited by initial endostatin therapy. However, additional studies are needed to verify this assumption.

Subgroup analysis showed a significant improvement or a trend toward improvement in PFS and OS with extended therapy in most subgroups, except for female patients and those with EGFR mutation. A possible reason for this discrepancy between female and male patients may be related to the small sample size of female patients in the control group (only 9 cases). However, the discrepancy between patients with and without EGFR mutation maybe attributed to the fact that in the control population, subsequent treatment with EGFR TKIs may provide a survival benefit that minimizes risk due to the short duration of endostatin + PT-DC treatment (less than 3 cycles).

Furthermore, we compared toxicity profiles between the extended therapy group and the control group. According to previous literature, systemic antiangiogenic therapy may result in systemic toxicity.^[30]^ In this study, patients treated with 4 or more cycles of endostatin plus PT-DC did indeed have higher rates of hematologic and gastrointestinal toxicities as compared with the control patients. Nevertheless, these adverse events were considered chemotherapy-related, and notably, there were no statistically significant differences in almost all grade 3 to 4 toxicities between the 2 treatment groups. The only apparent toxicity related to endostatin was cardiac disorder, but its incidence rate was similar in the 2 study arms. Additionally, most cardiac disorders were mild and did not interrupt the continuation of therapy. Taken together, these results support the safety of the extended use of endostatin plus PT-DC as a treatment for patients with advanced NSCLC.

Several limitations should be addressed here regarding the present study. First, the retrospective and nonrandomized nature of the study is its major limitation, which may have introduced inherent selection biases that could weaken the conclusions. Other limitations include the relatively small sample size and the use of data from a single institution, which might reduce the generalizability of our findings.

In conclusion, the current study indicates that the extended treatment of endostatin combined with PT-DC is able to provide further survival benefits with satisfactory toxicity in previously untreated patients with advanced NSCLC. Our findings support the use of endostatin as a maintenance therapy for NSCLC, which merits further evaluation in randomized trials.

## Acknowledgements

The authors thank Dr Zhenhuan Zheng for his kind assistance in data analysis and manuscript preparation. The authors also thank Beijing Municipal Administration of hospitals Clinical medicine Development of special funding (ZYLX201509) for the support.
